# The importance of health policy and systems research for strengthening rehabilitation in health systems: a call to action to accelerate progress

**DOI:** 10.1186/s12998-023-00519-8

**Published:** 2023-12-11

**Authors:** Walter R. Frontera, Wouter DeGroote, Abdul Ghaffar

**Affiliations:** 1 American Journal of Physical Medicine and Rehabilitation, University of Puerto Rico School of Medicine, San Juan, PR 00936-5067 Puerto Rico; 2 Rehabilitation Programme, World Health Organization, Geneva, Switzerland; 3 Bloomberg School of Public Health, Johns Hopkins University, Baltimore, MD USA

During the last few decades, the field of rehabilitation has experienced substantial development, growth, and acceptance. Rehabilitation addresses the impact of a health condition on a person’s everyday life by optimizing their functioning and reducing their experience of disability. Rehabilitation expands the focus of health beyond preventative and curative care to ensure people with a health condition can remain as independent as possible and participate in education, work and meaningful life roles [16]. A definition of rehabilitation for research purposes has been recently published [7]. Scientific and clinical research have generated a body of knowledge that strongly supports the use of many rehabilitation interventions with positive outcomes in various populations and health conditions.

We also have now a better understanding of the growing global need, demand, and recognition of rehabilitation around the world. For example, it has been estimated that 2.41 billion people in the world could benefit from rehabilitation services. This means that at least one in every three persons in the world needs rehabilitation at some point during the course of their disease or injury [2]. This figure has most likely increased due to the COVID-19 pandemic. The need for rehabilitation increased by 63% between 1990 and 2017 due to the aging population, the increasing prevalence of noncommunicable health conditions, and the shifting epidemiological profile in most countries [2]. Finally, according to the 2022 Global report on health equity for persons with disabilities, approximately 1.3 billion people or 16% of the world’s population has moderate to severe levels of disability associated with the underlying health conditions and impairments [13]. Now more than ever before, it is crucial that rehabilitation is available and accessible to populations globally according to their needs. The important contribution of rehabilitation to the functioning, including social and occupational participation and well-being of populations worldwide, can no longer be denied or delayed. Rehabilitation is critical for the attainment of the United Nations Sustainable Development Goal 3, *Ensure healthy lives and promote well-being for all at all ages* [9].

Notwithstanding the foregoing arguments, there continues to be a high unmet need for rehabilitation globally, with some low- and middle-income countries reporting unmet needs up to 50% of those who could benefit from rehabilitation. Rehabilitation services are not accessible to many people around the world [5]. Many of those in need do not have access because of the failure, at least partially, to effectively plan for rehabilitation services. Many nations and health systems have not implemented policy measures that recognize rehabilitation as an essential component of universal health coverage [6, 8]. Health policy, planning, and decision-making for rehabilitation often require more local evidence to adequately plan, finance, implement, and monitor quality rehabilitation services including infrastructure and workforce to make services accessible to those in need [12].

The field of health policy and systems research (HPSR) seeks to understand and improve how societies organize themselves in achieving collective health goals, and how different actors interact in the policy and implementation processes to contribute to policy outcomes [1, 10]. By nature, it is inter-disciplinary, a blend of economics, sociology, anthropology, political science, law sciences, public health, and epidemiology that together draw a comprehensive picture of how health systems respond and adapt to health policies, and how health policies can shape − and be shaped by − health systems and the broader determinants of health. The importance of HPSR for rehabilitation has been recently highlighted with robust data that needs to be considered and used by health policy and systems community and leadership [3]. HPSR for rehabilitation generates the evidence needed by policy makers to make appropriate decisions and to develop action plans to enhance the capacity of the health system to serve the population in need of rehabilitation services. For example, the evidence generated by HPSR helps to (1) establish priorities for rehabilitation service delivery, (2) evaluate outcomes of various rehabilitation interventions in relation to the levels of care in the health system, (3) identify specific benefits to society justifying those decisions, and (4) strengthen health systems to increase access, quality, and provision of health services for rehabilitation [4]. Supported by the recent resolution on ‘Strengthening rehabilitation in health systems’ that has been endorsed by the World Health Assembly for the first time in the history of the WHO [15], it is time to leverage HPSR to support societal health goals as they apply to rehabilitation.

In 2022 the WHO Rehabilitation Program established the World Rehabilitation Alliance (WRA) [14] to strengthen networks and partnerships that advocate for the integration of rehabilitation into health systems. The WRA is a WHO-hosted global network of stakeholders whose mission and mandate are to support the implementation of the Rehabilitation 2030 Initiative [11] through advocacy activities. The WRA focuses on promoting rehabilitation as an essential health service that is integral to Universal Health Coverage and to the realization of the United Nations Sustainable Development Goal 3. The work of the WRA is divided into five workstreams: workforce, primary care, emergencies, external relations, and research. The research workstream is dedicated to the generation and routine use of HPSR evidence for planning and integrating rehabilitation into health systems. The specific objectives of this workstream are to advocate for (1) the demand and utilization of HPSR evidence for rehabilitation, (2) the widespread generation of high-quality HPSR evidence for rehabilitation, and (3) the publication, dissemination, and implementation of HPSR evidence for rehabilitation.

In this context the co-authors of this editorial on behalf of their respective academic journals express their full support for the WRA mission in general and for the specific objectives of the research workstream. In concrete terms we, commit that our journals, as much as possible, will implement one or more of the following actions: (1) invite researchers in the field of HPSR for rehabilitation to submit their manuscripts to our Journals for peer review and possible publication, (2) create a special journal section, series, or designation dedicated to HPSR for rehabilitation, (3) appoint editorial board members with expertise in HPSR for rehabilitation, and (4) disseminate research articles among funding agencies and policymakers. These actions by our academic journals will help the WRA achieve its goal of strengthening rehabilitation services for all.
